# Phosphorylation of the multifunctional signal transducer B-cell adaptor protein (BCAP) promotes recruitment of multiple SH2/SH3 proteins including GRB2

**DOI:** 10.1074/jbc.RA119.009931

**Published:** 2019-09-16

**Authors:** Johannes U. Lauenstein, Atul Udgata, Alex Bartram, Delphine De Sutter, David I. Fisher, Samer Halabi, Sven Eyckerman, Nicholas J. Gay

**Affiliations:** ‡ Department of Biochemistry, University of Cambridge, Cambridge CB2 1GA, United Kingdom; § Department of Biomolecular Medicine, Ghent University, VIB Center for Medical Biotechnology, VIB, A. Baertsoenkaai 3, Ghent B-9000, Belgium; ¶ Discovery Sciences, Discovery Biology, IMED Biotech Unit, AstraZeneca, Cambridge CB4 0WG, United Kingdom

**Keywords:** Toll-like receptor (TLR), phosphorylation, Src homology 2 domain (SH2 domain), Src homology 3 domain (SH3 domain), signal transduction, B-cell adaptor protein, immunity, kinase signaling, phosphoinositide 3-kinase, virotrap

## Abstract

B-cell adaptor protein (BCAP) is a multimodular, multifunctional signal transducer that regulates signal transduction pathways in leukocytes, including macrophages, B-cells, and T-cells. In particular, BCAP suppresses inflammatory signaling by Toll-like receptors (TLRs). However, how BCAP itself is regulated and what its interaction partners are is unclear. Here, using human immune cell lines, including THP-1 cells, we characterized the complex phosphorylation patterns of BCAP and used a novel protein complex trapping strategy, called virotrap, to identify its interaction partners. This analysis identified known interactions of BCAP with phosphoinositide 3-kinase (PI3K) p85 subunit and NCK adaptor protein (NCK), together with previously unknown interactions of BCAP with Src homology 2 (SH2) and SH3 domain-containing adaptor proteins, notably growth factor receptor-bound protein 2 (GRB2) and CRK-like proto-oncogene, adaptor protein (CRKL). We show that the SH3 domain of GRB2 can bind to BCAP independently of BCAP phosphorylation status, suggesting that the SH2 domains mediate interactions with activated receptor tyrosine kinase complexes including the CD19 subunit of the B-cell receptor. Our results also suggested that the PI3K p85 subunit binds to BCAP via SH3 domains forming an inactive complex that is then activated by sequential binding with the SH2 domains. Taken together, our results indicate that BCAP is a complex hub that processes signals from multiple pathways in diverse cell types of the immune system.

## Introduction

Toll-like receptors (TLRs)^2^ recognize a wide range of microbial ligands as well as danger-associated self-molecules as part of the innate immune response in vertebrates (1). Inflammatory TLR signaling promotes microbial clearance and initiates the adaptive immune response. The function of TLRs has mainly been studied in myeloid cells, such as macrophages and dendritic cells. However, they are also widely expressed in lymphoid cells, where they play an important role in B and T lymphocytes. *In vitro*, TLR stimulation leads to B-cell proliferation and differentiation into antibody secreting cells (2). *In vivo*, TLR signaling contributes to T-independent antibody responses (3, 4) and autoimmune-related pathologies (5, 6).

In these B-cell pathologies, cross-talk has been observed between the B-cell receptor (BCR) and TLR pathways (7). However, the molecular mechanisms and pathways of TLR signaling in B-cells are not fully understood. TLR7 and TLR9 have been shown to signal from the same compartments as BCRs, where dual engagement of BCR and TLR receptor can occur mediated by nucleic acid antigens (8). Recently, it was proposed that synergistic BCR/TLR signaling may be part of a supercomplex controlling oncogenic signaling in two major subtypes of diffuse large B-cell lymphomas (DLBCL) (9).

These findings are substantiated by the fact that TLRs and BCR share a common pool of adaptor proteins and kinases. In particular, the tyrosine kinases SYK, LYN, and BTK play important roles in B-cell development and activation. All three kinases have also been associated with TLR signaling (10–12). Most TLR receptors and several adaptor proteins are reported to be tyrosine phosphorylated, although the kinases responsible and the functional importance remain unclear (13).

Adaptor proteins shared between the TLR and BCR pathway include TAK1 and DOCKS8, B-cell scaffold protein with ankyrin repeats (BANK1), and B-cell adaptor protein (BCAP) (10, 14, 15). Among these, BCAP is a versatile adaptor protein with roles in both myeloid and lymphoid cells, spanning multiple signaling pathways. It is a dimeric, multimodular protein with Toll/interleukin 1 receptor domains (TIRs) as well as ankyrin repeats, proline-rich regions, and canonical tyrosine phosphorylation motifs. In B-cells, BCAP links CD19 and cross-linked BCR to phosphoinositide 3-kinase (PI3K) signaling (16, 17). Additionally, this adaptor protein is implicated in calcium signaling as it associates with and controls phospholipase C-γ2 (PLC-γ2) activity (18). In T cells, BCAP couples PI3K activity and signaling by the interleukin 1 receptor (IL-1R), thereby regulating pathogenic Th17 cell differentiation (19). BCAP also negatively regulates TLR signaling via multitypic interactions with receptor and adaptor TIRs in macrophages, dendritic cells, and natural killer cells. This leads to the activation of PI3K (20, 21). The mechanism of negative regulation likely involves the metabolism of phosphatidylinositol lipids by PI3K and PLC-γ2 causing the depletion of the phosphatidylinositol (4,5)-diphosphate (PIP2) pool in the plasma membrane. The Mal/TIRAP adaptor protein binds to PIP2 and this interaction is necessary for signal transduction by the TLRs (23, 24).

Two key modes of interaction enable these regulatory functions of BCAP. First, three proline-rich regions facilitate SH3 domain interaction, with Nck1/2 as the single known binding partner (25). Second, ubiquitous tyrosine phosphorylation mediates SH2 domain interactions. Several studies have shown the importance of phosphorylation at Y*XX*M motifs in BCAP (16, 20, 21, 23, 26, 27). However, the kinases responsible for BCAP tyrosine phosphorylation in various cells types have not been fully determined. The kinase c-Abl phosphorylates BCAP at several non-Y*XX*M sites (28). Phosphorylation by c-Abl potentially provides binding sites for PLC-γ2, nonreceptor tyrosine kinases (PTKs), or novel SH2 domain containing interaction partners.

In chicken B-cells, SYK and to a lesser extent BTK contribute to BCAP tyrosine phosphorylation, whereas LYN-deficient B-cells showed an increase in BCAP phosphorylation (16). LYN was later found to be essential for BCAP phosphorylation downstream of mouse CD19, ectopically expressed in chicken B-cells (27). In this context, the absence of LYN could be rescued by a related Src kinase Fyn. Overexpression in HEK293T cells suggests that SYK plays a role in the phosphorylation of Y*XX*M motifs in BCAP (29). Later, it was shown that in HEK293T cells BCAP binds both SYK and LYN, indicative of LYN phosphorylation of BCAP (23, 27). In macrophages, however, SYK is not required for BCAP phosphorylation and association with PI3K (21). Given these somewhat conflicting reports, it remains unclear which kinases are responsible in which cellular context and organism. Moreover, the precise phosphorylation sites on BCAP, including the Y*XX*M motifs, are yet to be determined.

Our goal is to shed light on the distinct molecular pathways, associated adaptor proteins, and kinases that mediate the BCAP BCR and TLR cross-talk. Here we show that BTK, SYK, LYN, and casein kinases contribute to BCAP hyperphosphorylation. Using a virotrap protein interaction screen we have identified Grb2 as a novel BCAP-interaction partner that associates via SH3 domain interactions. Taken together with other proteins identified in the interaction screen, such as PI3K, Nck, CRKL, and casein kinases, our results reveal that BCAP functions as a complex immune signaling hub. We were able to map the interactions of Grb2 and several previously known BCAP partners at the single domain level, revealing a central role for SH2 and SH3 domain interactions.

## Results

### BCAP is hyperphosphorylated in mammalian cells

**Figure 1.**
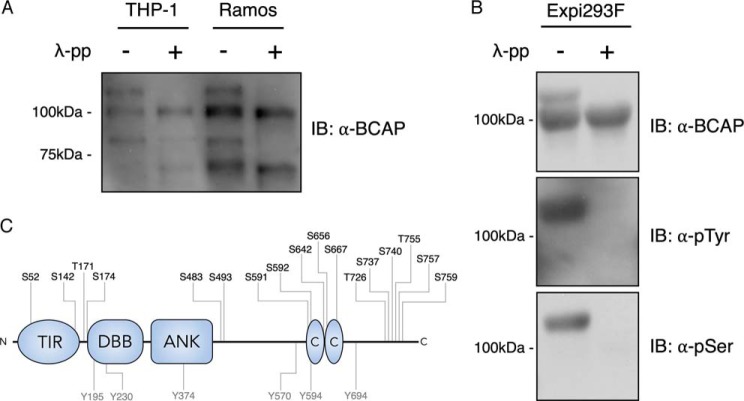
**BCAP is hyperphosphorylated in B-cell, macrophages, and Expi293F cells.** *A,* lysates from THP-1 and Ramos cells were dephosphorylated with λ-phosphatase and immunoblotted for BCAP. *B,* His-Avi-tagged BCAP expressed in Expi293F cells was purified and dephosphorylated with λ-phosphatase before immunostaining for tyrosine and serine phosphorylation. *C,* phosphorylation sites of BCAP expressed in Expi293F cells were determined by phosphopeptide mapping. BCAP was digested with trypsin, chymotrypsin, Asp-N, and Glu-C prior to MS.

To investigate the phosphorylation state of BCAP we used Western blotting to probe several cell types including macrophages and B-cells. In all cases, endogenous BCAP appears as multiple bands (Fig. 1*A*). The bands corresponding to the long splice isoform of BCAP (BCAP-L) extend from the expected molecular mass of around 90 kDa to greater than 100 kDa (16, 20, 26). Likewise, when BCAP is expressed in HEK293-derived Expi293F cells, a similar pattern is observed (Fig. 1*B*). Incubation of cell extracts with λ-phosphatase shows that the observed changes in electrophoretic mobility are caused entirely by phosphorylation (Fig. 1, *A* and *B*). Further Western blot analysis revealed that both tyrosine and serine/threonine-linked phosphates are present in BCAP. In addition, MS analysis of the BCAP protein purified from HEK293T cells identifies phosphorylated serine and threonine residues and six phosphotyrosines (Fig. 1*C*).

### BCAP is readily phosphorylated by BTK and to a lesser extent SYK and LYN

**Figure 2.**
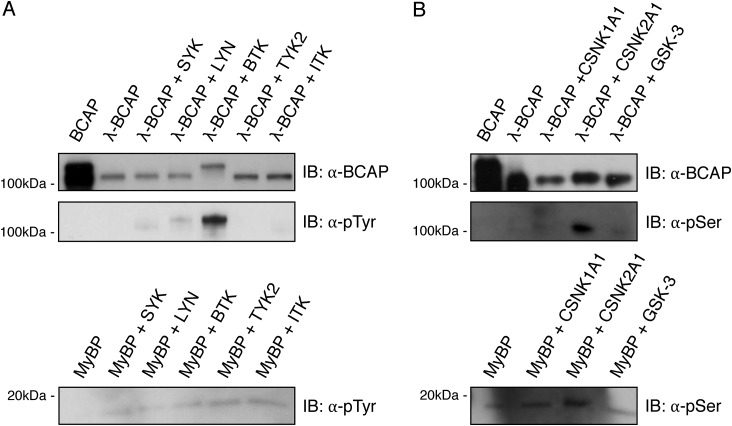
**BCAP is phosphorylated by BTK, LYN, and SYK.** Purified BCAP, dephosphorylated BCAP (λ-BCAP), and myelin basic protein (MyBP) were phosphorylated with (*A*) tyrosine kinases SYK, LYN, BTK, TYK2, and ITK, or (*B*) serine kinases CSNK1A1, CSNK2A2, and GSK-3.

To determine the kinases responsible for BCAP hyperphosphorylation, an *in vitro* kinase assay was performed using recombinant dephosphorylated BCAP. After λ-phosphatase treatment, purified BCAP was incubated with SYK, LYN, and BTK. Under these conditions, nonreceptor tyrosine kinases SYK, LYN, and BTK were able to phosphorylate BCAP, but BTK is by far the most efficient (Fig. 2*A*). Phosphorylation by SYK and LYN was substantially less relative to BTK although LYN phosphorylation of BCAP still resulted in a partial band shift when probed for tyrosine phosphorylation. To validate the specificity of the assay, other kinases were included in the experiments, but TYK2 and ITK, a member of the TEC kinases, were not able to phosphorylate BCAP under these conditions (Fig. 2*A*).

### Virotrap protein interaction screens identify novel BCAP-binding partners

**Figure 3.**
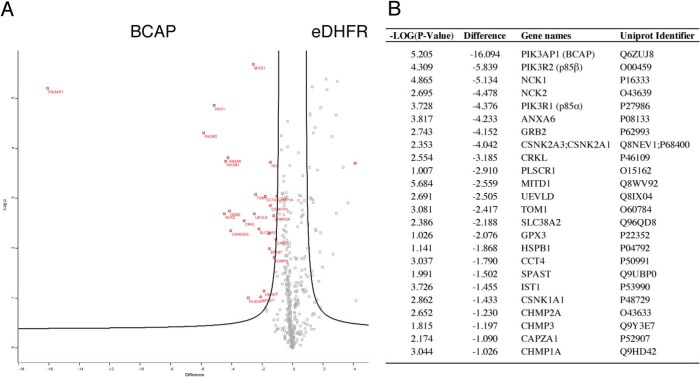
**Virotrap interaction screen reveals novel BCAP-interaction partners.** HEK293T cells were transfected with GAG-BCAP and a pMD2.G-pcDNA3-FLAG-VSV-G mix to generate FLAG-VSV-G–coated VLPs. After purification and tryptic digest, the VLP contents were analyzed by MS. *A,* volcano plot of BCAP VLP contents compared with the eDHFR control. False discovery rates (*FDR*) = 0.05 and S0 = 1. Proteins that are significantly enriched in either BCAP or eDHFR VLPs are highlighted in *red. B,* overview of significant BCAP virotrap hits sorted according to relative enrichment.

To further elucidate the interaction network of BCAP and to identify molecular mechanisms of BCR and TLR cross-talk, we performed a virotrap interaction screen (30). A fusion protein between the HIV-1 GAG protein and BCAP was expressed in HEK293T cells, resulting in the budding of virus-like particles (VLPs) that contain the BCAP bait construct as well as potential new interaction partners. Mass spectrometry analysis of the VLPs revealed three different groups of potential BCAP-interaction partners (Fig. 3). The first group includes the PI3K regulatory subunits p85 α/β and the adaptors Nck1 and -2, known binding partners of BCAP that interact via SH2 and SH3 domains. In addition, Grb2 and CRKL, SH2 and SH3 domain adaptor proteins that play a role in immunity and B-cells signaling were identified. The SH2 domain of Grb2 has previously been predicted to interact with BCAP based on sequence specificity (16). Additionally, previous MS-based interaction studies found BCAP in a Grb2 interaction screen, suggesting an SH3 domain-dependent association (31).

A second group of proteins detected by virotrap have not previously been linked to BCAP or BCR signaling. These include annexin A6, several variants of casein kinase (CSNK1/2), TOM1, and UEVLD. Annexins play a role in glucocorticoid-mediated innate immune responses and inflammation. Annexin A6 does not contain protein interaction domains that would explain a direct interaction with BCAP. However, other Annexin family proteins bind Grb2 (32). Casein kinases are ubiquitous serine and threonine kinases involved in numerous cellular functions including cell cycle progression, apoptosis, and transcription, as well as viral infection. Casein kinases phosphorylate substrates containing acidic residues C-terminal to the serine or threonine substrates. Indeed prediction algorithms indicate several residues in BCAP that are likely substrates of casein kinases and these residues are phosphorylated in BCAP purified from Expi293F cells (Table S1). Furthermore *in vitro* kinase assays show that CSNK2A1 but not CSNK1A1 or GSK-3 phosphorylate BCAP (Fig. 2*B*). It is therefore likely that CSNK2A1 contributes to the serine and threonine phosphorylation pattern found in BCAP. The role of these modifications remains unknown and further research is required to understand their mechanism of regulation.

The third group of proteins identified by virotrap are components of the ESCRT-III complex. As these have a role in viral budding, they are likely artifacts (30, 33). Notably, absent in the screen are TIR domain containing interaction partners of BCAP, such as MyD88, MAL, and SARM. This is likely due to the N-terminal GAG fusion blocking potential TIR domain interactions.

### Grb2 is a novel BCAP-interaction partner

**Figure 4.**
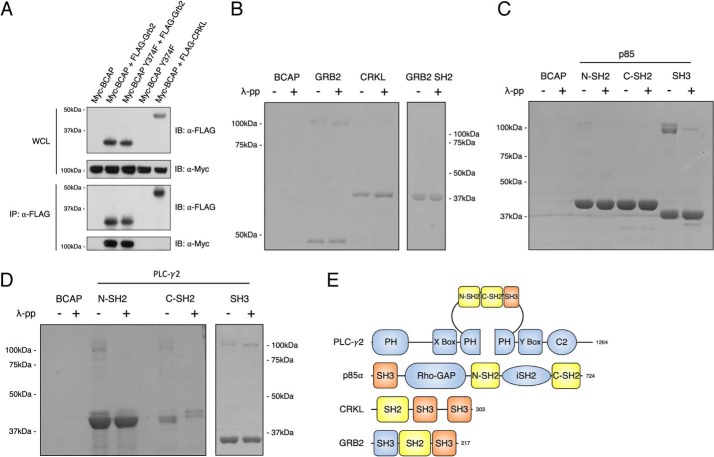
**BCAP engages in SH3 domain interaction and tyrosine phosphorylation-dependent SH2 domain interactions.** *A,* HEK293T cells were transfected with Myc-BCAP, Myc-BCAP Y374F, FLAG-GRB2, and FLAG-CRKL. At 24 h post-transfection, cells were lysed and subjected to immunoprecipitation with anti-FLAG antibody. Precipitates were split and immunostained for precipitation of FLAG-GRB2, FLAG-CRKL, and Myc-BCAP. *B,* purified GST-tagged GRB2, GRB2 SH2, CRKL; *C,* p85 N-SH2, p85 C-SH3, p85 SH3; *D,* PLC-γ2 N-SH2, PLC-γ2 C-SH2, and PLC-γ2 SH3 were immobilized on GST resin. Purified BCAP and dephosphorylated BCAP were subsequently applied to the resin and GST-tagged bait proteins were eluted from the resin and analyzed on SDS-PAGE. *E,* domain arrangement of BCAP-interacting proteins.

To further characterize the novel interaction partners identified in the virotrap screen, we expressed BCAP with Grb2 and CRKL in HEK293T cells. Co-immunoprecipitation experiments reveal that FLAG-Grb2 but not FLAG-CRKL co-immunoprecipitates with Myc-BCAP (Fig. 4*A*). This association was not dependent on the ^374^YPNT motif. These results show that the interaction of BCAP and Grb2 is likely direct, whereas that of CRKL is indirect. This seems plausible given that CRKL is known to bind the PI3K p85 subunit (34, 35). An advantage of the virotrap method is that it can detect multiple indirect interactions but the extent of these compared with direct binding in any screen is difficult to quantify (30).

**Figure 5.**
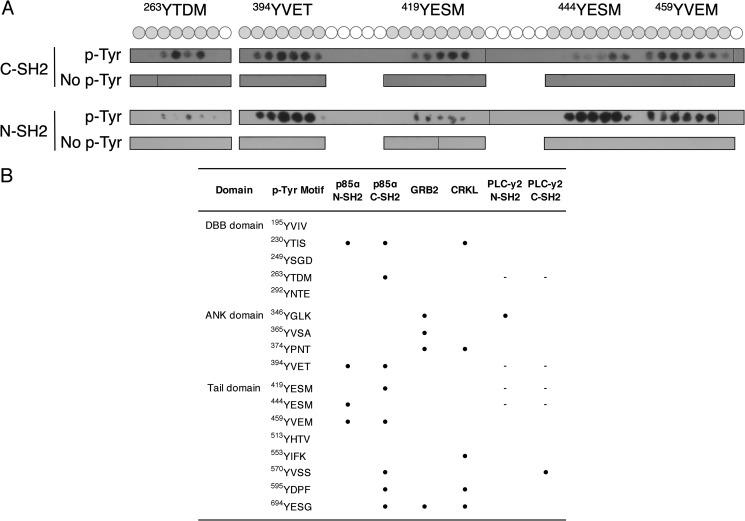
**Peptide arrays reveal binding sites for BCAP SH2 domain interactions.** Binding of SH2 domains to an array of 15-amino acid-long peptides containing BCAP tyrosine motifs. *A,* binding of p85 SH2 domains to BCAP Y*XX*M motifs. Phosphotyrosine containing peptides are depicted as a *gray circle. B,* binding of the SH2 domains of p85, GRB2, CRKL, and PLC-γ2 to phosphorylated tyrosine motifs in BCAP. The ● symbol indicates binding of the SH2 domain to a BCAP tyrosine motif. Motifs followed by “–” were not tested for the respective SH2 domain interaction.

To obtain domain level resolution of the interactions between BCAP and Grb2, CRKL, p85, and PLC-γ2, *in vitro* pulldown assays were performed. GST fusion proteins corresponding to full-length Grb2, but not to the Grb2 SH2 domain alone, co-purified with BCAP irrespective of its phosphorylation state (Fig. 4*B*). Thus, the SH3 domain rather than the SH2 domain mediates association of Grb2 with BCAP. Interestingly, this implies that the ^374^YPNT motif is not a binding site for the Grb2 SH2 domain even though peptide array data suggests that the SH2 domain interacts with this motif, and MS shows phosphorylation in HEK293T cells (Figs. 1*C* and 5*B*). This contradiction can be explained by the accessibility of the phosphotyrosine motif. A homology model of the BCAP ankyrin domain reveals that although Tyr-374 is somewhat exposed, the other amino acids of the motif form part of an α-helix that is buried in the core of the ankyrin-fold, and therefore not accessible for interaction with the SH2 domain (Fig. S1).

We have also investigated how BCAP binds to the PI3K p85 subunit and PLC-γ2. GST fusion proteins corresponding to individual SH2 and SH3 domains were used in pulldown assays with phosphorylated and dephosphorylated BCAP. This analysis confirms that the N-terminal SH2 (N-SH2) domain of p85 binds BCAP in a phosphorylation-dependent manner and that the SH3 domain binds strongly to both phospho-forms of BCAP (Fig. 4*C*). The N-SH2 interaction is expected as BCAP was initially characterized based on its affinity for the p85 N-SH2 domain (16). Failure to interact with the C-terminal SH2 domain (C-SH2) could be due to a lack of phosphorylation of all Y*XX*M motifs, or because this domain has a much lower affinity. The C-SH2 domain has a lower affinity for certain phosphotyrosine motifs than the N-SH2 domain (36). *In vivo*, this lower affinity of the C-SH2 could be compensated for because prior binding of the SH3 and N-SH2 domain enhances the affinity of C-SH2. PLC-γ2 appears to have a similar interaction mechanism to p85, with a robust interaction with the SH3 domain and N-SH2 domain, and somewhat weaker C-SH2 domain binding (Fig. 4*D*).

### Peptide array analysis reveals complex patterns of interactions by SH2 adaptor proteins with BCAP phosphotyrosine motifs

We have defined the interaction patterns of p85α, GRB2, CRKL, and PLCγ2 with BCAP using peptide arrays. In this analysis, libraries of peptides with either unmodified or phosphorylated tyrosine residues, corresponding to the sequence of the short BCAP isoform, were screened for binding by adaptor SH2 domains. This analysis revealed a promiscuous pattern of binding by both the p85α N-terminal and C-terminal SH2 domains with interaction sites identified in the DBB, ANK, and tail regions of BCAP (Fig. 5 and Fig. S2). By contrast PLC-γ2 bound to only two sites, Tyr-346 in the ankyrin repeats and Tyr-570 in the tail domain for the N- and C-terminal SH2 domains, respectively (Fig. 5*B*). Grb2 interacts with 4 phosphotyrosines. Three of these are in the ANK domain and do not overlap with the p85α-binding sites (Fig. 5*B*). Although direct interaction of CRKL is not observed in pulldown experiments (Fig. 4*B*) array analysis identifies 5 potential phosphotyrosine-binding sites dispersed throughout the BCAP sequence (Fig. 5*B*).

## Discussion

In all cell types we have studied, BCAP has multiple isoforms. We determined that phosphorylation is the basis of this phenomenon and identified that BCAP is the substrate of several kinases including tyrosine kinases BTK, SYK, and LYN. BCAP tyrosine phosphorylation is a central part of BCAP activation and has been observed downstream of TLR, IL-1R, and BCR signaling complexes (16, 19, 20). Deletion of tyrosine phosphorylation motifs results in loss of function phenotypes downstream of TLR4 activation, providing further evidence for the importance of tyrosine phosphorylation (21). BTK strongly phosphorylated BCAP in an *in vitro* kinase assay and caused a band shift equivalent to that seen *in vivo* (Fig. 1). Phosphorylation of BCAP by SYK and LYN was much weaker and may be directed to a smaller number of tyrosine motifs. Previous reports from Matsumura *et al.* (29) showed that SYK phosphorylation is mainly targeted toward three Y*XX*M motifs. *In vivo*, we expect a certain degree of redundancy between these kinases and the precise combination of kinases acting on BCAP is likely to vary depending on the context of the activated receptor, co-receptors, and adaptor proteins. Indeed, a previous study showed genetically that SYK signaling was not required for BCAP tyrosine phosphorylation or PI3K association in murine macrophages (21).

The virotrap experiments presented were carried out in the absence of innate stimulus, which indicates that the identified interaction partners bind to BCAP in the resting state. Interestingly control experiments using cells that express constitutively active TLR4 do not identify additional interacting proteins.^3^ This finding is consistent with a previous study that found PI3K pre-associated with cytosolic BCAP (21). Thus, BCAP complexes are preformed and poised for recruitment to the plasma membrane in response innate stimuli.

The appearance of casein kinases in the virotrap screen was unexpected, but with an *in vitro* kinase assay we were able to confirm CSNK2 BCAP phosphorylation, as predicted by a phosphorylation site prediction tool. Casein kinases have previously been shown to regulate PI3K signaling by phosphorylation of PTEN (37). This activation of PI3K signaling by CSNK2A1 is synergistic with GSK-3, which does not phosphorylate BCAP in our *in vitro* kinase assay (38). Casein kinases have also been linked to innate immunity, by phosphorylation of various proteins in the NF-κB pathway (30, 39, 40). Our results now present BCAP as a substrate of CSNK2A1, suggesting a novel point of regulation for these serine/threonine kinases.

Our virotrap screen also identified Grb2 as a novel interaction partner that binds BCAP most likely through an SH3 domain interaction. Grb2 has both SH2 and SH3 domains that link growth factor receptors to Ras signaling, but also plays a crucial role in TCR and BCR signaling (41). In previous MS-based studies of the Grb2 interactome, BCAP was reported to interact indirectly through PI3K (31). A combination of *in situ* and *in vitro* experiments now show that Grb2 and BCAP bind directly. Interestingly, the *Drosophila* BCAP orthologue Dof couples fibroblast growth factor signaling through Grb2, indicating evolutionary conservation of this pathway (42).

The interaction of Grb2 is solely dependent on the SH3 domain, leaving the SH2 domain available to bind activated receptor complexes. A similar role has been described for Nck1/2 that links BCAP to the BCR signalosome (25, 43). The BCAP-Grb2 interaction may now explain how the BCAP-PI3K complex is recruited to CD19, as Grb2 is known to bind with CD19 through its SH2 domain (44). Through a similar mechanism, BCAP might be recruited to various other activated signaling complexes including RTKs and the TCR complex.

**Figure 6.**
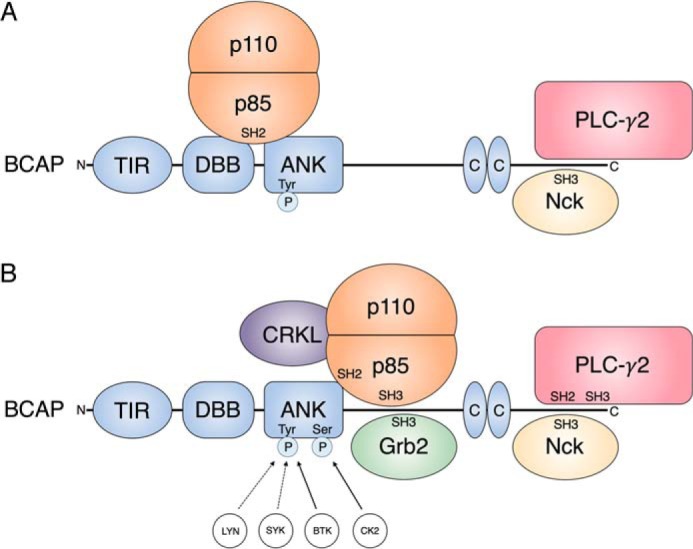
**Overview of the BCAP SH2 and SH3 domain interactome.** *A,* overview of the SH2 and SH3 domain-containing interaction partners of BCAP as described in the literature. *B,* updated BCAP interactome representing novel GRB2 and CRKL associations and detailing individual SH2 and SH3 domain interactions on BCAP. The model also includes kinases responsible for serine phosphorylation and tyrosine phosphorylation, which is required for SH2 domain interactions.

**Figure 7.**
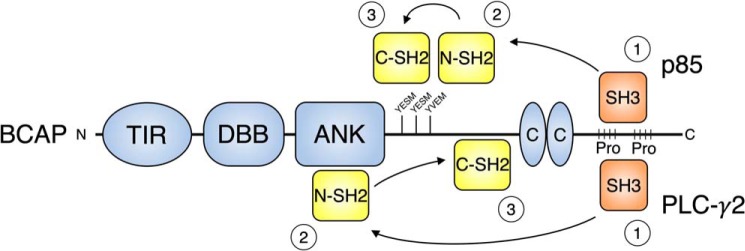
**Constitutive SH3 domain interactions facilitate rapid SH2 domain binding upon BCAP tyrosine phosphorylation.** Stepwise binding model for the SH2 and SH3 domain-containing BCAP-interaction partners p85 and PLC-γ2. The PI3K p85 or PLC-γ2 SH3 domains constitutively interact with BCAP proline-rich regions (*Pro*). The preformed complex can then rapidly engage in N-SH2 domain interaction upon BCAP tyrosine phosphorylation. High-affinity N-SH2 interactions facilitate the binding of lower-affinity C-SH2 domain interaction resulting in full activation of PI3K and PLC-γ2.

Our results also reveal the importance of other SH3 domain interactions of BCAP (Fig. 6). Interaction with PI3K p85 is in part mediated by the SH3 domain but weaker N-SH2 and C-SH2 domain interactions also contribute. Based on this information we expect that the constitutive binding of BCAP and p85 that was reported in macrophages and confirmed in our cell culture experiments is mediated by the p85 SH3 domain (21). Recruitment to activated receptor tyrosine kinases complexes would lead to phosphorylation of BCAP tyrosine motifs, enabling sequential N-SH2 and C-SH2 binding and PI3K activation (Fig. 7). P85 is known to bind tandem phosphotyrosine residues located a short distance apart (10–30 amino acids) (36, 45). Our peptide array data shows that the p85 SH2 domain binds to the three C-terminal Y*XX*M in BCAP that are arranged in this way (Fig. 5). For PLC-γ2 we expect a similar mechanism, where the SH3 domain is responsible for the constitutive interaction observed in HEK293T cells (Fig. 7) (23). In fact, sequential binding of the SH2 domains to release autoinhibition of PLC-γ2 has been reported (46).

## Experimental procedures

### Cell culture

THP-1 cells and Ramos (RA 1, ATCC) were maintained in RPMI 1640 medium (supplemented with 10% fetal bovine serum, l-glutamine, 100 units/ml of penicillin, and 100 mg/ml of streptomycin; all from Invitrogen). THP-1 cells were differentiated to macrophages using 10 ng/ml of phorbol 12-myristate 13-acetate (Sigma) for 12 h, followed by rest for 24 h in complete RPMI 1640 medium.

HEK293T cells (ATCC) maintained in Dulbecco's modified Eagle's medium (supplemented with 10% fetal bovine serum, l-glutamine, 100 units/ml of penicillin, and 100 mg/ml of streptomycin; all from Invitrogen). Expi293F cells (Thermo Fisher Scientific) were maintained in Expi293 Expression media at 37 °C, 8% CO_2_ and 140 rpm.

### Constructs

Constructs (47) for bacterial expression were generated by ligation-independent cloning (48) into pMCSG10 plasmid (DNASU) containing a His-GST-TEV_cl_ tag (Table S2). For expression in mammalian cells, plasmids were cloned using restriction enzymes and primer sequences contained the respective tags (Table S2). The pMET7-GAG-PQS1-RAS plasmid used in the cloning of pMET7-GAG-BCAP was derived from pMET7-GAG-RAS (Addgene number 80604) containing a unique quantification peptide as previously described (47).

### Virotrap

Virotrap experiments were performed essentially as described (30) with a minor modification in the transfection conditions and using an alternative data analysis strategy. In brief, 10 million authenticated HEK293T cells were seeded the day before transfection in T75 bottles. After 24 h cells were transfected with 6.4 μg of pMET7-GAG-BCAP bait construct, or with 3.75 μg of a pMET7-GAG-eDHFR control construct. For single step purification, a one-half ratio of expression vectors for VSV-G and FLAG-VSV-G was co-transfected in the cells (for a total of 1.1 μg). Transfection mixtures were normalized with mock vector (pSVsport). The supernatants containing Virotrap particles were harvested 40 h after transfection and cleared by centrifugation and filtering (0.45 μm). Supernatants were then incubated with MyOne Streptavidin T1 beads loaded with BioM2 antibody. Two h after binding, beads containing Virotrap particles were washed, and particles were released by competition with FLAG-peptide. After removal of the beads, samples were processed with Amphipols and digested using trypsin. After acidification, peptides were analyzed by LC-MS using a Thermo Scientific Q Exactive hybrid quadrupole-Orbitrap mass spectrometer. Analysis was performed on three independent transfections for BCAP and control experiments.

### MS analysis, peptide identification, data visualization, and phosphopeptide mapping

The peptide mixtures were first loaded on a trapping column (made in-house, 100 μm inner diameter × 20 mm, 5-μm beads, C18 Reprosil-HD, Dr. Maisch, Ammerbuch-Entringen, Germany). After flushing from the trapping column, the sample was loaded on an analytical column (made in-house, 75 μm inner diameter × 150 mm, 5-μm beads, C18 Reprosil-HD, Dr. Maisch) packed in the nanospray needle (PicoFrit SELF/P PicoTip emitter, PF360-75-15-N-5, New Objective). The samples were loaded and separated with a linear gradient from 98% solvent A′ (0.1% formic acid in water) to 40% solvent B′ (0.08% formic acid in water/acetonitrile, 20/80 (v/v)) in 30 min at a flow rate of 300 nl/min. This was followed by a 15-min wash reaching 99% solvent B′. The Q Exactive instrument was operated in data-dependent, positive ionization mode, automatically switching between MS and MS/MS acquisition for the 10 most abundant peaks in a given MS spectrum. The source voltage was 3.4 kV, and the capillary temperature was 275 °C. One MS1 scan (*m*/*z* 400–2000, AGC target 3 × 10^6^ ions, maximum ion injection time 80 ms) acquired at a resolution of 70,000 (at 200 *m*/*z*) was followed by up to 10 tandem MS scans (resolution 17,500 at 200 *m*/*z*) of the most intense ions fulfilling the defined selection criteria (AGC target 5 × 10^4^ ions, maximum ion injection time 80 ms, isolation window 2 Da, fixed first mass 140 *m*/*z*, spectrum data type: centroid, minimum AGC target 1000, intensity threshold 1.4 × 10^4^, exclusion of unassigned, 1, 5–8, and >8 charged precursors, peptide match preferred, exclude isotopes on, dynamic exclusion time 12 s). The higher collision dissociation energy was set to 25% normalized collision energy and the polydimethyl cyclosiloxane background ion at 445.120025 Da was used for internal calibration (lock mass).

All MS data were searched using MAXQUANT (version 1.5.7.4) against the human SwissProt database (Jan 2017; complemented with GAG, VSV-G, and eDHFR sequences) with 4.5 and 20 ppm tolerance on precursor and fragment mass, respectively, with trypsin/P settings allowing up to two missed cleavages and with methionine oxidation and N-terminal acetylation formation as variable modifications. Minimum peptide length was set to 7, and maximum peptide mass was 4600 Da. PSM FDR and protein FDR were set to 0.01. Minimum peptides and minimum razor + unique peptides were set to 1. The searches were performed together with the corresponding control samples to allow matching of MS spectra between runs. Contaminants and identifications against the REVERSE database were removed in the PERSEUS (version 1.5.5.3) analysis after the log2 transformation of the non-normalized protein LFQ ratios. Only proteins identified in all three samples of the BCAP or eDHFR conditions were retained for further analysis. Default PERSEUS settings were used for missing value imputation from a normal distribution. A two-sided *t* test was performed with multiple testing correction using 1000 randomizations. FDR was set at 5% with the S0 curve set at 1.

For phosphopeptide mapping, BCAP purified from Expi293F cells and dephosphorylated BCAP were separated on SDS-PAGE. Digestion with trypsin, chymotrypsin, Asp-N, Glu-C, and subsequent MS was performed at the CCPcore MS facility (University of Cambridge, Department of Biochemistry) and the VIB Proteomics Core (VIB, Ghent). The MS proteomics data have been deposited to the ProteomeXchange Consortium via the PRIDE partner repository (22)(data set identifier to be confirmed).

### Co-immunoprecipitation in HEK293T cells

For transfection, HEK293T cells were transfected around 70–80% confluence using JetPEI (Polyplus Transfection SA) according to the manufacturer's recommendation. 3 μg of plasmid DNA was transfected per well in a 6-well plate, and when required pcDNA3.1 was to ensure 3 μg of DNA was transfected. 24 h after transfection, cells were washed with PBS and lysed with 300 μl of Tris immunoprecipitation buffer (20 mm Tris, 150 mm NaCl, 0.5% Nonidet P-40, 1 mm EDTA, pH 8.0), which was supplemented with 50 mm NaF, 5 mm orthovanadate, 60 mm β-glycerophosphate, and 1× protease inhibitor mixture (Calbiochem). The lysate was incubated for 30 min at 4 °C with agitation. After centrifugation, the supernatant was collected for Western blotting or immunoprecipitation with anti-FLAG M2 beads (F2426, Sigma) according to the manufacturer's recommendation. The whole cell lysate and immunoprecipitation samples were analyzed by Western blotting using anti-FLAG M2 (F3165, Sigma), anti-Myc (9B11, Cell Signaling Technology), and anti-mouse IgG-horseradish peroxidase (A9044; Sigma).

### Protein expression and purification

Recombinant His- or GST-tagged proteins were expressed in *Escherichia coli* strain Rosetta2 (DE3) (Merck Chemicals) and subsequently purified from soluble lysates. His-tagged proteins were lysed in Tris lysis buffer (50 mm Tris, 150 mm NaCl, and 30 mm imidazole, pH 7.5). For purification Ni-NTA beads were used and His-tagged proteins were eluted in lysis buffer containing 500 mm imidazole. GST-tagged proteins were lysed in PBS and purified with GSH beads, and eluted with 10 mm reduced GSH. After affinity purification, His- and GST-tagged proteins were subjected to size exclusion with a HiLoad 16/600 Superdex 200-pg column equilibrated in size-exclusion buffer (20 mm Tris, 150 mm NaCl, 1 mm tris(2-carboxyethyl)phosphine, and 5% glycerol, pH 7.5).

His-Avi-TEV_cl_-BCAP (FL) was expressed in Expi293F cells. Cells were transfected at a density of 4 × 10^6^ cells/ml with 1.5 μg/ml of plasmid DNA and 6 μg/ml of linear PEI Max (Polysciences). 24 h post-transfection, fresh medium was added to the culture to double the volume and cells were harvested 3 days post-transfection by centrifugation. For lysis, cell pellets were resuspended in Tris lysis buffer, supplemented with 50 mm NaF, 5 mm orthovanadate, 60 mm β-glycerophoaphate, and 1× protease inhibitor mixture (Calbiochem). After affinity purification using Ni-NTA beads, the sample was treated with TEV protease to remove the His-Avi tag. Part of the sample was simultaneously treated with λ-protein phosphatase (p0753; New England Biolabs) according to the manufacturer's recommendations. After affinity purification proteins were subjected to size exclusion with a HiLoad 16/600 Superdex 200 pg column equilibrated in size-exclusion buffer buffer.

### GST Pulldown assays

For *in vitro* pulldown assays, 50 μg of GST-Grb2 and GST-CRKL, and 100 μg of GST-Grb2-SH2, GST-p85-N-SH2, GST-p85-C-SH2, GST-p85-SH3, GST-PLC-γ2-N-SH2, GST-PLC-γ2-C-SH2, and GST-PLC-γ2-SH3 were loaded onto 100 μl of GSH beads equilibrated with PBS. After washing the samples with PBS, 50 μg of BCAP (FL) and dephosphorylated BCAP (FL) were applied to the column. Following three wash steps with PBS, samples were eluted using 10 mm reduced GSH and analyzed on SDS-PAGE.

### Peptide array

Peptide arrays spanning the BCAP Y*XX*M motifs and other tyrosines were synthesized on cellulose membranes (JPT Peptide Technologies). Arrays were activated in methanol and blocked in a solution of 2.5% BSA and 0.05% Tween 20 in TBS (TBS-T). Arrays were then incubated with 5 μg/ml of GST-p85-N-SH2, GST-p85-C-SH2, GST-Grb2, GST-CRKL, GST-PLC-γ2-N-SH2, or GST-PLC-γ2-C-SH2 overnight and transferred to nitrocellulose blotting membranes via sequential blotting according to the manufacturer's recommendation. Bound proteins were detected by chemiluminescence with anti-GST (MA4–004; Thermo Fisher Scientific) followed by anti-mouse IgG-HRP (A9044; Sigma).

### Kinase assay

For the *in vitro* kinase assays, 2 μg of dephosphorylated BCAP (FL) or 100 μg of dephosphorylated myelin basic protein (31314; Active Motif) were diluted in 500 μl of kinase buffer (50 mm HEPES, 10 mm MgCl_2_, 0.01% BRIJ35, 1 mm EGTA, and 150 μm ATP, pH 7.5). Upon adding 60 pmol of SYK (PV3857; Thermo Fisher Scientific), LYN (PV6448; Thermo Fisher Scientific), BTK (PV3363; Thermo Fisher Scientific), TYK2 (PV4790; Thermo Fisher Scientific), ITK (PV4193; Thermo Fisher Scientific), CSNK1A1 (PV3850; Thermo Fisher Scientific), or CSNK2A1 (PV3248; Thermo Fisher Scientific), the samples were incubated at 30 °C for 30 min. The reaction was stopped using 4× SDS loading dye, and the samples were analyzed using Western blotting. For chemiluminescence detection, anti-BCAP (AF4857; R&D Systems), anti-phosphotyrosine (Ab179530; Abcam), anti-phosphoserine (Ab9332, Abcam), anti-rabbit IgG-HRP (A0545; Sigma), and anti-goat IgG-HRP (A5720; Sigma) were used.

## Author contributions

J. U. L. and N. J. G. conceptualization; J. U. L. data curation; J. U. L. and S. E. formal analysis; J. U. L., S. E., and N. J. G. supervision; J. U. L., A. U., A. B., D. D. S., D. I. F., S. H., and S. E. investigation; J. U. L. and N. J. G. writing-original draft; S. E. resources; S. E. methodology; S. E. and N. J. G. project administration; N. J. G. funding acquisition; N. J. G. writing-review and editing.

## Supplementary Material

Supporting Information

## Footnotes

3 N. Gay and J. Lauenstein, unpublished data.

## Footnotes

2 The abbreviations used are:

TLR: Toll-like receptor
BCAP: B-cell adaptor protein
SH2: Src homology domain 2
SH3: Src homology domain 3
PI3K: phosphoinositide 3-kinase
PLC-γ: phospholipase Cγ
GRB2: growth factor receptor-bound protein 2
VLP: virus-like particle
BCR: B-cell receptor
TIR: Toll/interleukin 1 receptor domain
IL: interleukin
PIP2: phosphatidylinositol (4,5)-diphosphate
HRP: horseradish peroxidase
TEV: tobacco etch virus
GSK-3: glycogen synthase kinase-3
N-SH2: N-terminal SH2
C-SH2: C-terminal SH2
Ni-NTA: nickel-nitrilotriacetic acid
DHFR: dihydrofolate reductase
GST: glutathione *S*-transferase
FDR: false discovery rate
VSV: vesicular stomatitis virus.
