# Supplementary material for: Phosphorylation of the multifunctional signal transducer B-cell adaptor protein (BCAP) promotes recruitment of multiple SH2/SH3 proteins including GRB2
Source: J Biol Chem. 2019 Sep 16;294(52):19852–61. doi: 10.1074/jbc.RA119.009931 (PMC6937578; doi:10.1074/jbc.RA119.009931)
Supplement: Supporting Information [file supp_294_52_19852__index.html]

Phosphorylation of the multi functional signal transducer B-cell adaptor protein (BCAP) promotes recruitment of multiple SH2/SH3 proteins including GRB2 — BCAP phosphorylation and inflammatory signalling — Phosphorylation of the multifunctional signal transducer B-cell adaptor protein (BCAP) promotes recruitment of multiple SH2/SH3 proteins including GRB2 — BCAP phosphorylation and inflammatory signalling — Supporting Information 

# Phosphorylation of the multifunctional signal transducer B-cell adaptor protein (BCAP) promotes recruitment of multiple SH2/SH3 proteins including GRB2

## Supporting Information

- Supporting Information (to be published online) - Supplementary Figures (2) and Tables (2)
