# Supplementary material for: Phosphorylation of the multifunctional signal transducer B-cell adaptor protein (BCAP) promotes recruitment of multiple SH2/SH3 proteins including GRB2
Source: J Biol Chem. 2019 Sep 16;294(52):19852–61. doi: 10.1074/jbc.RA119.009931 (PMC6937578; doi:10.1074/jbc.RA119.009931)
Supplement: Supporting Information [file supp_RA119.009931_153607_1_supp_393923_pxq5yp.pdf]

SUPPORTING INFORMATION

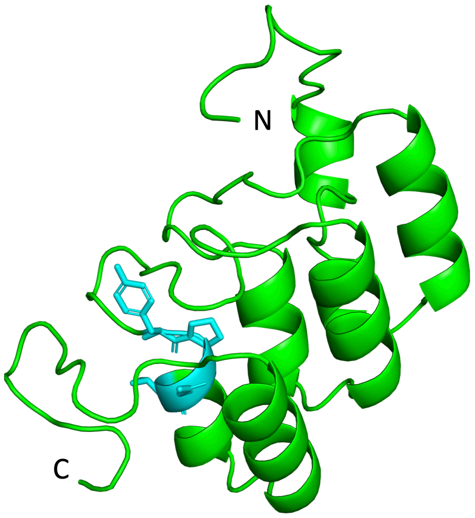

Supplementary Figure 1. The 374YPNT motif is buried in the BCAP ANK domain.
Structural model of the BCAP ANK domain generated by the Phyre2 prediction server. Tyrosine 374 and the remaining residues of the 374YPNT motif are highlighted in teal and represented as sticks.

A

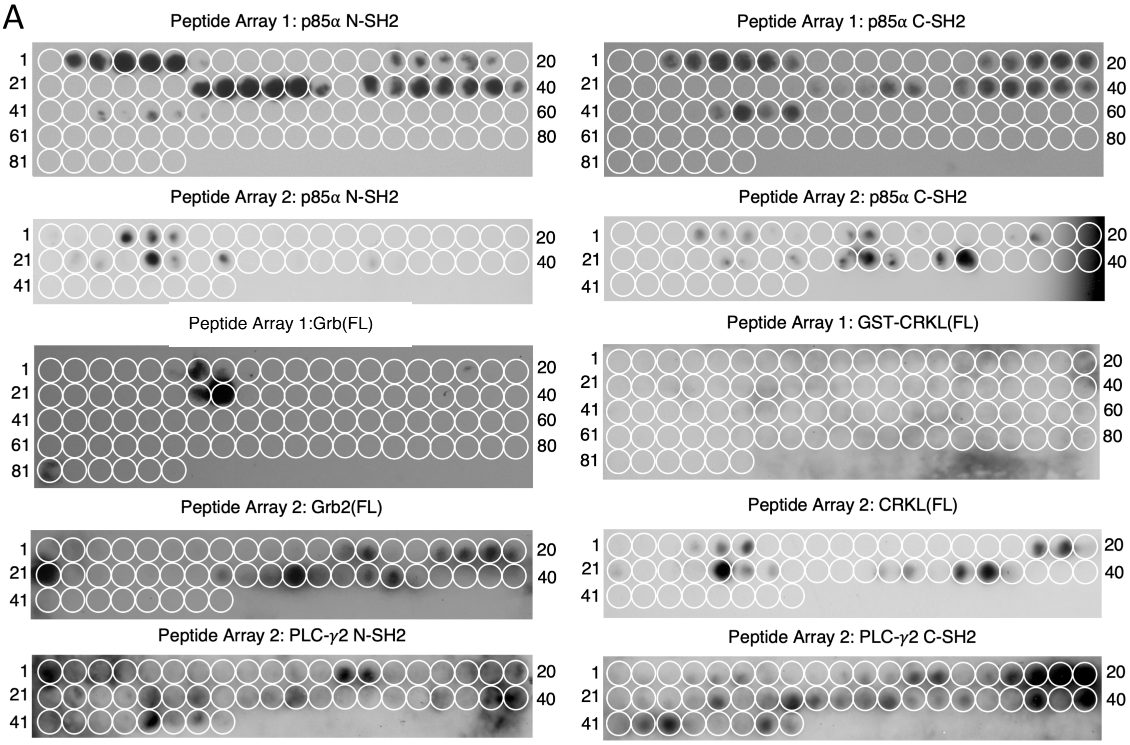

B Peptide Array 1

1 KHGFRDLRQFIDE-pY-V	23 LSTDLLMKCSLNPGC	45 ETVISY-pY-TDMEEIGN	67 LNPGCDEDLYESMAA
2 GFRDLRQFIDE-pY-VET	24 TDLLMKCSLNPGCDE	46 VCETVISY-pY-TDMEEI	68 PGCDEDLYESMAAFV
3 RDLRQFIDE-pY-VETVD	25 LLMKCSLNPGCDEDL	47 SY-pY-TDMEEIGNLLSN	69 CDEDLYESMAAFVPA
4 LRQFIDE-pY-VETVDML	26 MKCSLNPGCDEDL-pY-E	48 -pY-YTDMEEIGNLLSNAA	70 EDLYESMAAFVPAAT
5 QFIDE-pY-VETVDMLKS	27 CSLNPGCDEDL-pY-ESM	49 DMEEIGNLLSNAANP	71 LYESMAAFVPAATED
6 IDE-pY-VETVDMLKSHI	28 LNPGCDEDL-pY-ESMAA	50 KHGFRDLRQFIDEYV	72 ESMAAFVPAATEDLY
7 E-pY-VETVDMLKSHIKE	29 PGCDEDL-pY-ESMAAFV	51 GFRDLRQFIDEYVET	73 MAAFVPAATEDLYVE
8 VETVDMLKSHIKEEL	30 CDEDL-pY-ESMAAFVPA	52 RDLRQFIDEYVETVD	74 AFVPAATEDLYVEML
9 TVDMLKSHIKEELMH	31 EDL-pY-ESMAAFVPAAT	53 LRQFIDEYVETVDML	75 VPAATEDLYVEMLQA
10 DMLKSHIKEELMHGE	32 L-pY-ESMAAFVPAATED	54 QFIDEYVETVDMLKS	76 AATEDLYVEMLQAST
11 LKSHIKEELMHGEEA	33 ESMAAFVPAATEDL-pY-	55 IDEYVETVDMLKSHI	77 TEDLYVEMLQASTSN
12 SHIKEELMHGEEADA	34 MAAFVPAATEDL-pY-VE	56 EYVETVDMLKSHIKE	78 DLYVEMLQASTSNPI
13 IKEELMHGEEADAV-pY-	35 AFVPAATEDL-pY-VEML	57 IKEELMHGEEADAVY	79 YVEMLQASTSNPIPG
14 EELMHGEEADAV-pY-ES	36 VPAATEDL-pY-VEMLQA	58 EELMHGEEADAVYES	80 GDLVVCETVISYYTD
15 LMHGEEADAV-pY-ESMA	37 AATEDL-pY-VEMLQAST	59 LMHGEEADAVYESMA	81 LVVCETVISYYTDME
16 HGEEADAV-pY-ESMAHL	38 TEDL-pY-VEMLQASTSN	60 HGEEADAVYESMAHL	82 VCETVISYYTDMEEI
17 EEADAV-pY-ESMAHLST	39 DL-pY-VEMLQASTSNPI	61 EEADAVYESMAHLST	83 ETVISYYTDMEEIGN
18 ADAV-pY-ESMAHLSTDL	40 -pY-VEMLQASTSNPIPG	62 ADAVYESMAHLSTDL	84 VISYYTDMEEIGNLL
19 AV-pY-ESMAHLSTDLLM	41 EMLQASTSNPIPGDG	63 AVYESMAHLSTDLLM	85 SYYTDMEEIGNLLSN
20 -pY-ESMAHLSTDLLMKC	42 GDLVVCETVISY-pY-TD	64 YESMAHLSTDLLMKC	86 YTDMEEIGNLLSNAA
21 SMAHLSTDLLMKCSL	43 LVVCETVISY-pY-TDME	65 MKCSLNPGCDEDLYE	
22 AHLSTDLLMKCSLNP	44 VCETVISY-pY-TDMEEI	66 CSLNPGCDEDLYESM	

Peptide Array 2

1 IRCGAETTV-pY-VIVRC	13 PTLLHFAAK-pY-GLKNL	25 AHQLPDNEP-pY-IFKVF	37 GAETTVYVIVRCKLD
2 GAETTV-pY-VIVRCKLD	14 HFAAK-pY-GLKNLTALL	26 LPDNEP-pY-IFKVFAEK	38 KVENEYTISVKAPNL
3 TV-pY-VIVRCKLDDRVA	15 AK-pY-GLKNLTALLLTC	27 EP-pY-IFKVFAEKSQER	39 NVSLKIYSGDLVVCE
4 RMEAKVENE-pY-TISVK	16 LTCPGALQA-pY-SVANK	28 KSQERPGNF-pY-VSSES	40 AFKIVPYNTETLDKL
5 KVENE-pY-TISVKAPNL	17 PGALQA-pY-SVANKHGH	29 ERPGNF-pY-VSSESIRK	41 HFAAKYGLKNLTALL
6 NE-pY-TISVKAPNLSSG	18 QA-pY-SVANKHGHYPNT	30 NF-pY-VSSESIRKGPPV	42 PGALQAYSVANKHGH
7 SSGNVSLKI-pY-SGDLV	19 YSVANKHGH-pY-PNTIA	31 WRDRPQSSI-pY-DPFAG	43 ANKHGHYPNTIAEKH
8 NVSLKI-pY-SGDLVVCE	20 ANKHGH-pY-PNTIAEKH	32 RPQSSI-pY-DPFAGMKT	44 GQEEDVYHTVDDDEA
9 KI-pY-SGDLVVCETVIS	21 GH-pY-PNTIAEKHGFRD	33 SI-pY-DPFAGMKTPGQR	45 LPDNEPYIFKVFAEK
10 MCQAFKIVP-pY-NTETL	22 CHLGQEEDV-pY-HTVDD	34 LPAKVEFGV-pY-ESGPR	46 ERPGNFYVSSESIRK
11 AFKIVP-pY-NTETLDKL	23 GQEEDV-pY-HTVDDDEA	35 KVEFGV-pY-ESGPRKSV	47 RPQSSIYDPFAGMKT
12 VP-pY-NTETLDKLLTES	24 DV-pY-HTVDDDEAFSVD	36 GV-pY-ESGPRKSVIPPR	48 KVEFGVYESGPRKSV

Supplementary Figure 2. Complete BCAP binding profiles of the p85, Grb2, CRKL and PLC-γ2 SH2 domains.
(A) Peptide arrays were incubated with GST-tagged SH2 domains as indicated. Protein binding was visualised by immunoblotting. (B) Sequence overview of peptide arrays used in (A). For completeness this representation includes repeats of arrays showing interaction of p85 SH2 domains presented in Figure 5A.

Supplementary Table 1. KinasePhos CSNK2 prediction BCAP of phosphorylation.

Position	Target Residue	Kinase	Sequence	SVM score
52	S	CSNK2	GPEA**S**FSAE	0.562
54	S	CSNK2	EASF**S**AEDL	0.879
72	S	CSNK2	VVLL**S**AELV	0.549
149	S	CSNK2	SGCD**S**VTDT	0.586
213	S	CSNK2	EAEF**S**PEDS	0.887
592	S	CSNK2	RPQS**S**IYDP	0.730
720	S	CSNK2	TDST**S**STAS	0.653
740	S	CSNK2	LSVS**S**GMEG	0.895

Supplementary Table 2. Overview of constructs

Construct	Domain boundaries	Cloning method	Template	Vector
Grb2 SH2	86-179	LIC	pGEX Grb2	pMCSG10
FLAG-Grb2	1-217	HindIII and XhoI	pGEX Grb2	pcDNA3.1(+)
FLAG-CRKL	2-303	BamHI and XhoI	pGEX-2T CRKL	pcDNA3.1(+)
p85 N-SH2	359-478	LIC	Myc-p85α	pMCSG10
p85 C-SH2	652-758	LIC	Myc-p85α	pMCSG10
p85 SH3	39-121	LIC	Myc-p85α	pMCSG10
PLC-γ2 N-SH2	534-634	LIC	PLC-γ2-V5	pMCSG10
PLC-γ2 C-SH2	649-734	LIC	PLC-γ2-V5	pMCSG10
PLC-γ2 SH3	769-829	LIC	PLC-γ2-V5	pMCSG10
Myc-BCAP Y374F	1-805	SDM	Myc-BCAP	pcDNA3.1(+)
His-Avi-TEV_{cl}-BCAP	2-805	HindIII and BamHI	Myc-BCAP	pcDNA3.1(+)
pMET7-GAG-BCAP	1-805	EcoRI and XbaI	Myc-BCAP	pMET7-GAG-PQS1-RAS
